# Characterization of herpes simplex virus clinical isolate Y3369 as a glycoprotein G variant and its bearing on virus typing

**DOI:** 10.1186/1743-422X-8-290

**Published:** 2011-06-09

**Authors:** Daniel N Clark, Brian D Poole, Daniel V Hammond, Tyler J Hedman, Danny S Catts, Amanda Stewart, F Brent Johnson

**Affiliations:** 1 Department of Microbiology and Molecular Biology, Brigham Young University, Provo, UT, 84602, USA; 2 Richards Laboratories, Inc., Pleasant Grove, UT 84062, USA

**Keywords:** Herpes Simplex Virus, serotyping, glycoprotein G

## Abstract

**Background:**

Herpes simplex viruses exist as two major serotypes, type 1 (HSV-1) and type 2 (HSV-2). Determination of type, either HSV-1 or HSV-2, is important in accurate diagnosis and clinical control of transmission. Several tests are available for typing HSV, including a monoclonal antibody specific for glycoprotein G and several PCR assays.

**Findings:**

A clinical isolate was identified as herpes simplex virus, but tested negative for both HSV-1 and HSV-2 antigens using type-specific monoclonal antibody assays. The isolate was determined to be HSV-1 by PCR analysis. A mutation which likely caused the monoclonal antibody non-reactivity was found in glycoprotein G. Phylogenetic analysis revealed two groups of HSV, one with the mutation and one without. Three population studies examining mutations in HSV-1 glycoprotein G were analyzed by chi-squared test. To this point, the epitope which the monoclonal antibody recognizes was only found in HSV-1 isolates from human European populations (*p *< 0.0001).

**Conclusions:**

These findings suggest that the PCR-based methods for HSV typing may be more useful than the standard monoclonal antibody test in areas of the world where the variant in glycoprotein G is more prevalent.

## Findings

Herpes simplex viruses exist as two major serotypes, type 1 (HSV-1) and type 2 (HSV-2). Determination of type, either HSV-1 or HSV-2, is important in accurate diagnosis and clinical control of transmission. Tests which can determine HSV type include viral antigen tests, serological tests of human antibodies and PCR [1,2]. The importance of glycoprotein G as the test analyte is emphasized by the 2002 STD Treatment Guidelines from the CDC: "Accurate type-specific assays for HSV antibodies must be based on the HSV-specific glycoprotein G2 for the diagnosis of infection with HSV-2 and glycoprotein G1 for diagnosis of infection with HSV-1." [3].

A clinical sample of a herpes simplex virus, designated Y3369 was isolated and proved refractory to typing. The isolate was obtained from an infected genital tract of a 48-year-old female patient. It was submitted to Richards Laboratories, Inc., Pleasant Grove, Utah, USA for diagnostic workup. The sample was incubated overnight, and then stained for virus-infected cells using a type-common polyclonal primary antibody and visualized by the immunoperoxidase technique using a rapid culture method [4,5]. The culture showed an abundance of cells positive for antibody labeling and had HSV-typical cytopathic effects, confirming the presence of HSV in the specimen (results not shown).

**Figure 1:**
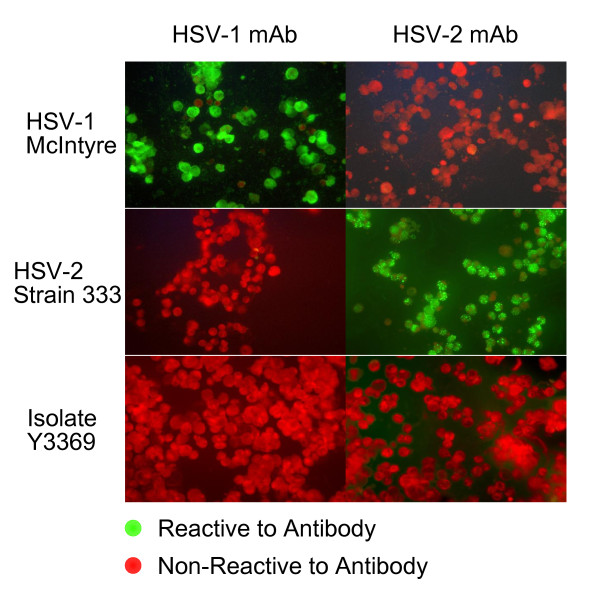
**Non-reactivity of strain Y3369 to HSV-1 and HSV-2 monoclonal antibodies**. MV1Lu cells infected with known HSV types (HSV-1 strain McIntyre; HSV-2 strain 333) and clinical isolate Y3369 were examined for reactivity of type-specific monoclonal antibodies (mAb). Negative reactivity is indicated by the red Evan's blue counterstain. Infection of C1008 cells yielded similar results.

The Y3369 isolate was then tested using the Wampole type-specific viral antigen test for HSV glycoprotein G. A viral stock culture was generated by inoculation of a portion of the rapid culture isolate into a culture of MV1Lu cells (mink lung, ATCC CCL-64). The specimen was also incubated in C1008 cells (Vero subline, ATCC CRL-1586) and subjected to similar serotypic analysis by staining with virus-specific monoclonal antibodies (mAbs) against HSV type 1 and type 2. These tests failed to yield a positive identification of the isolate as either HSV-1 or HSV-2 using type-specific mAb assays (Wampole Laboratories). The immunofluorescence result was negative against both reagent antisera in MV1Lu cells (Figure 1). The virus was also untypable in C1008 cells (not shown). The laboratory strains HSV-1 McIntyre and HSV-2 strain 333 were tested with mAb reagents and expected monotypic results were observed in these controls.

**Figure 2:**
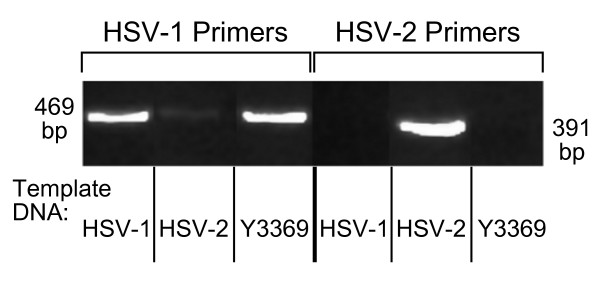
**Isolate Y3369 is an HSV-1 strain, not an HSV-2 virus by PCR of the HSV pol gene**. Viral DNA was isolated and the pol gene region was amplified by PCR using primers specific for either the HSV-1 or the HSV-2 sequence. PCR results were electrophoresed and compared between the Y3369 isolate and the McIntyre HSV-1 strain and the 333 HSV-2 strain. Image was edited to change lane order for ease of comparison.

Determination of HSV type was done by PCR specific for the HSV pol gene using a common forward primer and type-specific reverse primers as performed by Abraham, et. al [6] and Kimura, et al. [7]. DNA was extracted (Invitrogen PureLink viral DNA/RNA mini kit) from purified virus of HSV-1 (McIntyre strain), HSV-2 (Strain 333), and from the Y3369 isolate. PCR products were then analyzed on a 1% agarose gel (Figure 2), which revealed that clinical isolate Y3369 contains the pol gene of an HSV-1 virus. To confirm the analysis, DNA was then extracted from the gel (QIAquick gel extraction kit, Qiagen) and sequenced (Parallab 350, ABI 3730xl). DNA sequencing confirmed Y3369 specimen was a strain of HSV-1 with the sequenced amplicon having 100% identity when compared to the published HSV-1 pol gene sequence (GenBank accession #X04771) and only 85% homology with the HSV-2 sequence. Confirmation of the isolate as an HSV-1 strain was done by successful PCR amplification of HSV-1 genes UL1, UL10, UL22, glycoprotein D, and glycoprotein G (data not shown, see Table 1 for PCR conditions and primers).

**Table 1:** PCR condistions and Primers

Primer	Sequence
UL1 for	5'-GAGACCCCCTCGGCTATAAA-3'
UL1 rev	5'-CGTTTCTGTTTCCTGGGTGT-3'
UL10 for	5'-GAGCCTTGTGGGCACTTATG-3'
UL10 rev	5'-GTGATCTGCAGCAACCAAGA-3'
UL 22 for	5'-AAACAAAAGCGCTCCTCGTA-3'
UL22 rev	5'-GACAGACCCATGGTTTTTGG-3'
glycoprotein G for	5'-GCTGTTTGCGGGTTGGCACA-3'
glycoprotein G rev	5'-TCCCCCGCCCCATACCCTAC-3'
glycoprotein D for	5'-TTTGTGTGGTGCGTTCCGGT-3'
glycoprotein D rev	5'-TCCCATCCCAACCCCGCAGA-3'

PCR conditions for all reactions:

94°C 2 min, 40 cycles of [94°C 15 s, 57°C 15 s, 72°C 1:15], 72°C 2 min

**Figure 3:**
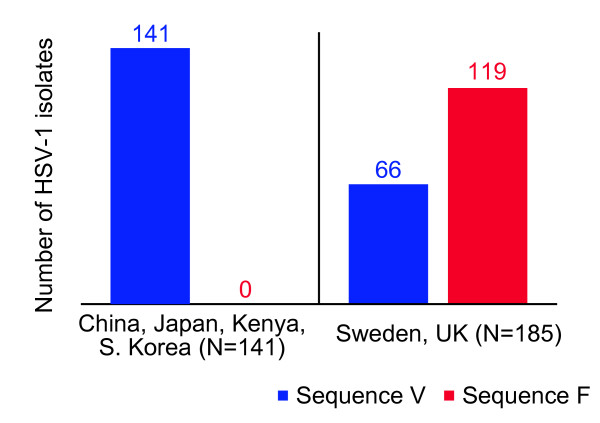
**Two groups of glycoprotein G are unequally distributed**. Of 185 isolates taken from Sweden and the UK, 64% contain sequence F, and this 64% represents the isolates which would properly type using the mAb which recognizes A**F**PL. By contrast, 100% of the 141 isolates taken from people in outside Europe, but only 36% of the European isolates contained the **v**aline at position 111 (see sequences in Figure 4). These sequences differ statistically by location using χ^2^, *p *< 0.0001.

Glycoprotein G was PCR amplified (see supplementary table) and sequenced. Examination of the sequences showed that the probable cause for the non-reactivity of the mAb assay was the presence of a valine residue in glycoprotein G at amino acid (AA) 111. This valine is near the immunodominant region of antibody binding during normal immune response [8]. Sequencing results were deposited [GenBank:HQ833203], and compared to other isolates on GenBank. Sequencing revealed that the clinical isolate Y3369 contains an amino acid sequence consistent with a common HSV-1 sequence found in many parts of the world [9] (Figure 3).

A meta analysis of three population studies which have sequenced this region of the HSV-1 US4 gene was conducted to determine the prevalence of valine at position 111, as was identified in our sample [9-11]. Included were isolates from individuals from China, Japan, Kenya, South Korea, Sweden, and the United Kingdom. We discovered the valine at position 111 to be present in all HSV-1 isolates (100%, N = 141) taken from human populations from Asia and Africa. The other populations, from the UK and Sweden, contained the valine at position 111 in 36% (N = 185) of isolates (Figure 3). This valine at position 111 is located within the binding site for a commonly used typing mAb, which recognizes the epitope AFPL [10]. The phenylalanine is replaced to form the sequence AVPL in this variant.

**Figure 4:**
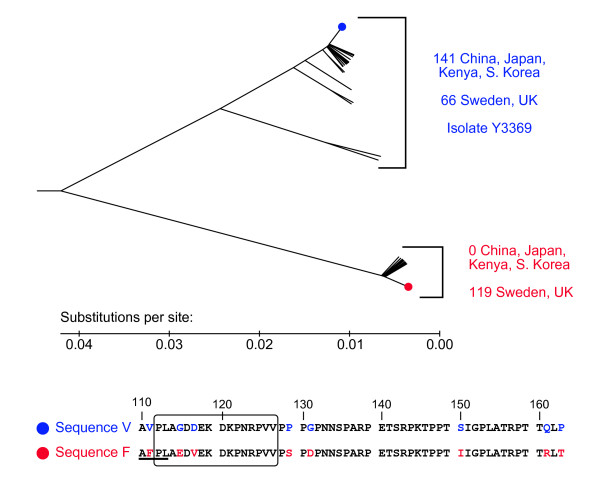
**Phylogenetic analysis reveals two groups of variations within HSV-1 glycoprotein G by region**. The evolutionary history was inferred using the UPGMA method. The tree is drawn to scale, with evolutionary distances in the units of base substitutions per site. There were a total of 165 positions, coding for AA 110 to 164. Phylogenetic analyses were conducted in MEGA4 [15]. Labeled branches represent the number of isolates from the specified region. Sequences shown at the bottom are from the branches highlighted with colored dots, which represent the most common sequence. Positions where sequences V and F differ are in color. The immunodominant region of glycoprotein G is enclosed by a box. The epitope AFPL (underlined, present exclusively in sequence F) is recognized by a common anti-glycoprotein G-1 mAb. The clinical isolate Y3369 discussed herein contains sequence V.

Sequences for the middle region encoding AA 110 to 164 of glycoprotein G were analyzed and a phylogenetic tree created (Figure 4). Phylogenetic analysis groups our isolate Y3369 as an HSV-1 with sequence V (representing valine at 111) which contains the sequence AVPL instead of AFPL, as well as other common nucleotides as shown in Figure 4. All isolates from populations from Africa and Asia, as well as 36% of the European population contained the sequence AVPL, which would not be recognized by the mAb which tests for the AFPL epitope. Another study found that all isolates with a valine residue at position 111 of glycoprotein G were untypable when assaying viral antigens [10]. This specific test would not be likely to function diagnostically in these African or Asian populations.

Our analysis of these studies provides evidence that glycoprotein G variation is likely significant in clinical typing discrepancies and also in isolate variations. Analysis of the amino acid sequences of Y3369 and other isolates indicates that there is a shared significant variation among HSV-1 strains that alters viral antigen assay specificity. PCR analysis is likely to succeed in HSV typing where the isolate is not recognized by the monoclonal antibody. In addition to results presented here, PCR has been used to type HSV samples on other occasions. In one study, 75 HSV-positive isolates yielded two which were untypable using type-specific antibody tests, later confirmed HSV-1 by PCR [12]. Another study yielded 1 untypable isolate of 37 tested HSV-positive isolates, which was also confirmed as HSV-1 by PCR [13]. These represent about 2% of the HSV-positive isolates in these two studies.

We have determined the presence of two phylogenetic groups of glycoprotein G. One group was only found in Europe (Figure 4, sequence F), and all the isolates in this group contain the epitope AFPL, which a common assay uses to type HSV-1. The other group (Figure 4, sequence V) was found in all tested regions, which include Africa, Asia and Europe. This group was characterized by the AVPL sequence. Y3369 is a member of this group. The two sequences differ by location statistically (χ^2 ^= 142.8, *p *< 0.0001).

The identification of these two groups, as well as their localization to different parts of the world, may aid in developing strategies for clinical viral antigen assays for HSV typing. Although the isolates included in the meta analysis which have the AVPL sequence were not tested by us, they would likely fail to type as HSV-1 using this same test. It should be considered that tests for the viral antigen epitope AFLP be used with caution in Africa or Asia.

This variation may also alter the interaction of virus with host. The presence of the variations in the immunodominant region of the protein suggests these mutations could be a result of viral immune evasion. These mutations may also affect the functioning of glycoprotein G, which involves attachment and entry [14]. Further tests are being performed to study what other effects this mutation has on the virus's efficiency of infection.

## List of abbreviations used

AA: amino acid; HSV: herpes simplex virus; mAb: monoclonal antibody;

## Competing interests

The authors declare that they have no competing interests.

This work was funded by Brigham Young University.

## Authors' contributions

DC was involved in experimental design and data acquisition, performed data analysis, conducted the meta analysis, and wrote the manuscript and figures. BP designed the study, participated in data analysis, and aided in writing and reviewing the manuscript. DH designed the study, performed PCR, gels, and sequencing; and was involved in writing the manuscript. TH, DK and AS all participated in data acquisition and writing the manuscript. FBJ performed antigen testing, conceived of the study, participated in study design, and participated in manuscript writing. All authors have read and do approve the final manuscript.

## Human Subjects

The specimen was submitted to a clinical laboratory for diagnostic workup by code number only and the work was performed under clinical laboratory licensure: Richards Laboratories of Utah, Inc., 55 East Center St., Pleasant Grove, UT 84062, Laboratory Director: Dr. F. Brent Johnson
